# Biomechanical effects of internal fixation with self-lock compression anti-rotation blade for Pauwels type III femoral neck fractures: a comparative finite element analysis

**DOI:** 10.1186/s12891-023-06386-x

**Published:** 2023-04-14

**Authors:** Bo-Xuan Huang, Si-Zheng Zhan, Ming Yang, Dian-Ying Zhang

**Affiliations:** 1 Department of Orthopedics and Trauma, Peking University People’s Hospital, No. 11 Xizhimen South Street, Xicheng District, Beijing, 100044 China; 2 Key Laboratory of Trauma and Neural Regeneration (Peking University), Ministry of Education, Beijing, 100044 China; 3 National Center for Trauma Medicine, Beijing, 100044 China

**Keywords:** Femoral neck fracture, Pauwels type III, Self-lock compression anti-rotation blade, Cannulated screw, Internal fixation, Finite element analysis

## Abstract

**Background:**

Self-lock compression anti-rotation blade (SCAB) is a novel internal fixation implant for femoral neck fractures (FNF). We conducted this finite element analysis study to evaluate the biomechanical performances of SCAB combined with a cannulated screw for fixation of Pauwels type III FNF.

**Methods:**

Three finite element models of Pauwels type III FNF treated with various internal fixations were established: a: the inverted triangular parallel cannulated screw (3CS) model, b: the biplane double-supported screw fixation (BDSF) model, c: the SCAB combined with a cannulated screw model. Displacement and Von Mises stress of femurs and internal fixations under increasing loads as well as the average stress on fracture surfaces and maximum displacements on the X and Z axis of proximal fracture fragments at maximum load were measured and compared.

**Result:**

The SCAB-based internal fixation exhibited superior biomechanical performances compared with 3CS and BDSF configurations, as the former resulted in lower parameters including displacement of the femur, Von Mises stress of internal fixation, stress on fracture surfaces as well as X and Z axis displacement of fracture fragments.

**Conclusion:**

Internal fixation using SCAB combined with a cannulated screw for Pauwels type III FNFs shows enough stability, with satisfied resistance to varus and shearing forces, which may provide a new option for the treatment of FNFs.

## Introduction

Pauwels type III femoral neck fractures (FNF) secondary to high-energy trauma are common in the young, and close reduction and internal fixation is the main surgical method to preserve patients’ hip joint [1, 2]. However, vertical Pauwels type III FNFs are unstable due to shearing forces [3] and after internal fixation the fracture fragments tend to displace, resulting in a high incidence of complications. According to the literature, the incidence of non-union after internal fixation of Pauwels type III FNF ranges from 16% to over 30%, and the incidence of femoral head necrosis from 11% to over 45%, seriously obstructing the prognosis of patients [4–6].

There are several internal fixation devices for treating FNFs such as cannulated screw (CS), dynamic hip screw (DHS), femoral neck system (FNS), and medial buttress plate. Among them, the method of internal fixation with cannulated screws has the advantages of minimal invasion, less destruction of bone, good preservation of blood supplies, and fewer costs [7, 8]. The traditional way of fixation with CS is parallelly driving three partially threaded cannulated screws (3CS) in an inversed triangle configuration into the femoral neck, but its mechanical stability has been found inferior to that of DHS, FNS, and medial buttress plates [1, 9–11]. To improve the performance of the internal fixation, many researchers focused on modifying the configurations of CS. Through a series of finite element analysis, Li et al. [12, 13] found that replacing two partial threaded screws in the triangular configuration with full threaded screws has better stability in the treatment of unstable femoral neck fractures. And the biomechanical effects of this hybrid screw fixation were also recognized by biomechanical tests performed on cadaveric bone by Cuellar et al. [14]. For Asian population with a smaller dimension of femoral neck, Chantarapanich et al. [15] suggested that the posterior triangular configuration presented biomechanical performance comparable to the conventional inverted triangle. Different from various triangular configurations, the biplane double-supported screw fixation method (BDSF, also called F-technique) reported by Filipov [16–18] was considered as an effect method of internal fixation of femoral neck fractures in non-elderly patients (age < 65 years). However, there are some technical difficulties of BDSF in clinical practice, which may cause unexpected damage to patients during operation. To provide an alternative option for the fixation of FNF, we designed a novel implant named the self-lock compression anti-rotation blade (SCAB) and obtained the patent (Patent number: ZL200710121931.7) [19]. Our previous study has demonstrated that SCAB is mechanically reliable and has the advantages of being minimally invasive, anti-rotation, of high pull-out strength, and no loss of bone during implantation [20].

We hypothesize that one SCAB combined with one cannulated lag screw (SCAB + CS) can provide sufficient stability for the fixation of FNFs. Hence, we aimed to compare and evaluate the mechanical performance of SCAB + CS, 3CS and BDSF for the internal fixation of vertical Pauwels type III FNFs by finite element analyses.

## Materials and methods

### Femoral neck fractures model establishment

The femur computer tomography (CT) data were obtained from a 30-year-old healthy male volunteer without any musculoskeletal disorders, from whom informed consent was obtained, using a Siemens 128-row CT scanner with a layer thickness of 1 mm. The CT image was stored in Digital Imaging and Communications in Medicine (DICOM) format. In the software Mimics 21.0 (Materialise, Leuven, Belgium), a three-dimensional model of the femur was reconstructed based on the CT image. Subsequently, a transcervical cutting plane at an angle of 40° with the horizontal plane was created in Mimics and the Pauwels type III femoral neck fracture model was established. Since there was no displacement between the two fracture fragments, it could be considered that the fracture had been reduced.

### Building the internal fixation model

**Fig. 1:**
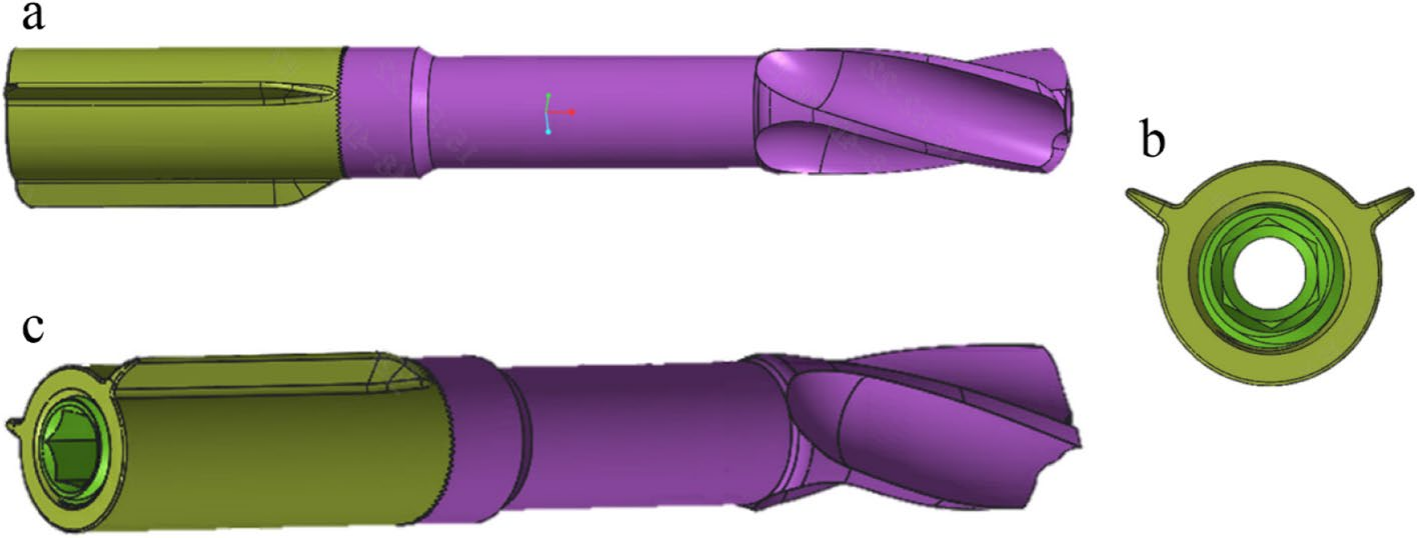
Diagram of SCAB (**a** Top view, **b** End view, **c** Oblique view) The design concept of the self-lock compression anti-rotation blade was partly based on the proximal femoral nail anti-rotation (PFNA). The helical blade and the connective bolt are the same as those in PFNA. In addition, we designed a sleeve part with denti-stripe for self-locking as well as compression and added two longitudinal straight tail fins arranged in a “V”-shape for anti-rotation. The SCAB is cannulated so it can be guided by a Kirschner wire

Models of partially threaded cannulated screw and SCAB were fabricated using Solidworks 2018 software (Dassault Systemes, Waltham, USA). The model of cannulated screw was reconstructed in 7.3 mm thread diameter, 16 mm thread length and 4.8 mm screw diameter. As in this research the threaded part of CS had little influence on the analytical results, the structure of thread was simplified, instead as a cylinder of equal diameter [21, 22]. The model of SCAB was made based on real geometrical dimensions (total length 95 mm, helical blade diameter 10 mm, helical blade length 24.8 mm, body diameter 9 mm, and the sleeve part diameter 10 mm) [20]. The diagram of SCAB was shown in Fig. 1. Then, using the software ANSYS 2021R1 (ANSYS, Canonsburg, USA), the fracture and implant models were assembled into three configurations described as below.

#### The 3CS model (Model A)

According to the literature [23], three cannulated screws parallel to each other were arranged in an inverted triangle configuration and placed in the femoral neck. The angles of the screws with the longitude axis of the femur were 135°, and the screw tip was 5 mm below the bone cortex of femoral head.

#### BDSF model (Model B)

According to the surgical method reported previously [17], three partially threaded cannulated screws were needed. First, the distal screw was placed in a posterior–proximal direction to touch onto the femoral calcar, the entry of which was 6 cm below the greater trochanter of femur and the angle of which was 160° with the femoral shaft. The second screw entered at 3 cm proximally from the distal one and the third one entered at 2 cm proximally from the middle one. Both latter two screws were parallelly directed anterior-proximal and with an angle of 135° towards the diaphysis of the femur.

#### SCAB + CS model (Model C)

The distal screw was implanted in a way similar to that of the distal screw in 3CS. Then a SCAB was parallelly placed at 2 cm proximally from the distal screw. The tips of SCAB and CS were 5 mm below the cortical bone of the femoral head.

**Fig. 2:**
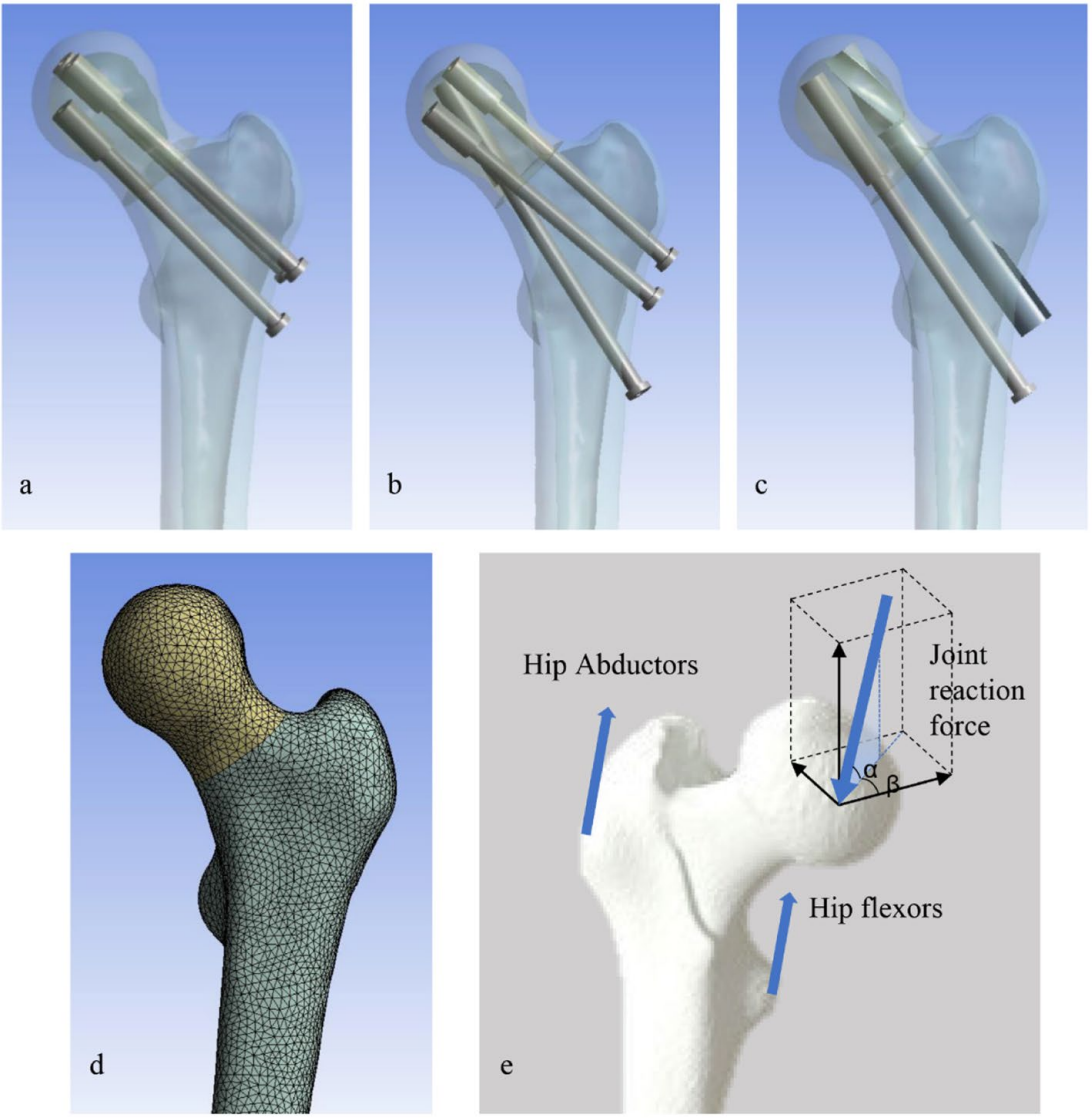
Finite element models of Pauwels type III FNF with internal fixations: (**a**) the inverted triangular parallel cannulated screw (3CS) model, (**b**) the biplane double-supported screw fixation (BDSF) model, (**c**) the SCAB combined with cannulated screw (SCAB + CS) model, (**d**) a sample mesh for the fractured bone, (**e**) details of applied loads of proximal femur

Assembled bone-implant models are shown in Fig. 2. Subsequently, models were imported into Ansys software for meshing and analyses.

### Material parameter settings

The fracture model was assumed to be anatomically repositioned. The bone model was assumed to be homogeneous, and isotropic with linear elastic materials. The implants were made of titanium alloy. The material property of the femur was set to represent young people. The properties of various materials are shown in Table 1. The relationship between bone and surfaces of helical blade and tail fins of SCAB as well as the threaded part of CS was set as tie constraints. The interfaces between bone and the body part of SCAB or CS were considered as friction, with a friction coefficient of 0.3 [24]. The friction coefficient between the fracture surfaces was set to be 0.46 [25].

**Table 1:** Material properties of bone and internal fixation

	Elastic modulus (GPa)	Poisson’s ratio
Cortical bone	16.8	0.3
Cancellous	0.84	0.2
Titanium alloy	105	0.35

### Boundary conditions and loading settings

Three forces in different directions were applied to proximal femur (Fig. 2). The joint reaction force was applied to the center of the femoral head at an angle of 13° laterally with the axis of femoral shaft in the coronal plane and an angle of 8° posteriorly in the sagittal plane, with a maximum value of 2100 N, corresponding to three times the body weight, simulating the force on the hip joint during the stance phase of walking [26, 27]. A force with an angle of 24° laterally in the coronal plane and 15° posteriorly in the sagittal plane was applied at the tip of the greater trochanter to simulate the load of the hip abductors with a maximum value of 1700 N. The force of hip flexors was applied on the lesser trochanter with an angle of 41° in the coronal plane and 26° in the sagittal plane with a maximum value of 770 N [28, 29]. The simulation process in this study was divided into four steps, with loading forces increasing from low to high (Table 2), aiming to simulate the rehabilitation process of postoperative patients from partial to complete weight-bearing. All nodes on the distal femoral surface were constrained with 0 degrees of freedom to prevent rigid body motion during analysis.

**Table 2:** Details of applied loads on the proximal femur models by steps

	Step 1	Step 2	Step 3	Step 3	Angle α	Angle β
Joint reaction force	500N	1000N	1500N	2100N	77°	8°
Hip abductors	425N	850N	1275N	1700N	24°	15°
Hip flexors	190N	380N	570N	770N	41°	26°

### Main outcome measures

The displacement distributions of femurs and the Von Mises stress distributions of internal fixations were examined. The averages of Von Mises stress on the fracture surfaces at maximum load were reported. In addition, the horizontal (X-axis) and vertical (Z-axis) displacements of proximal femoral fragments at maximum load were measured respectively.

## Result

### Displacement distributions of femurs

**Fig. 3:**
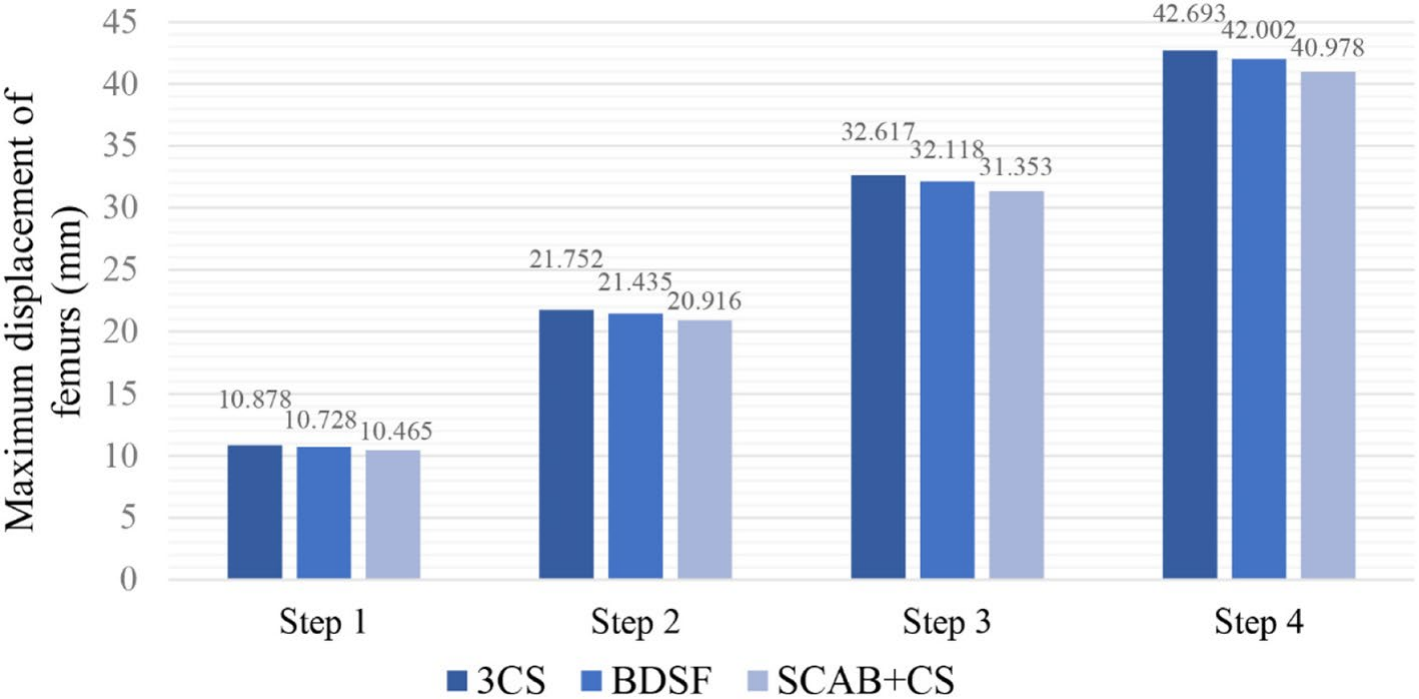
Maximum displacements of femurs under increasing loads

**Fig. 4:**
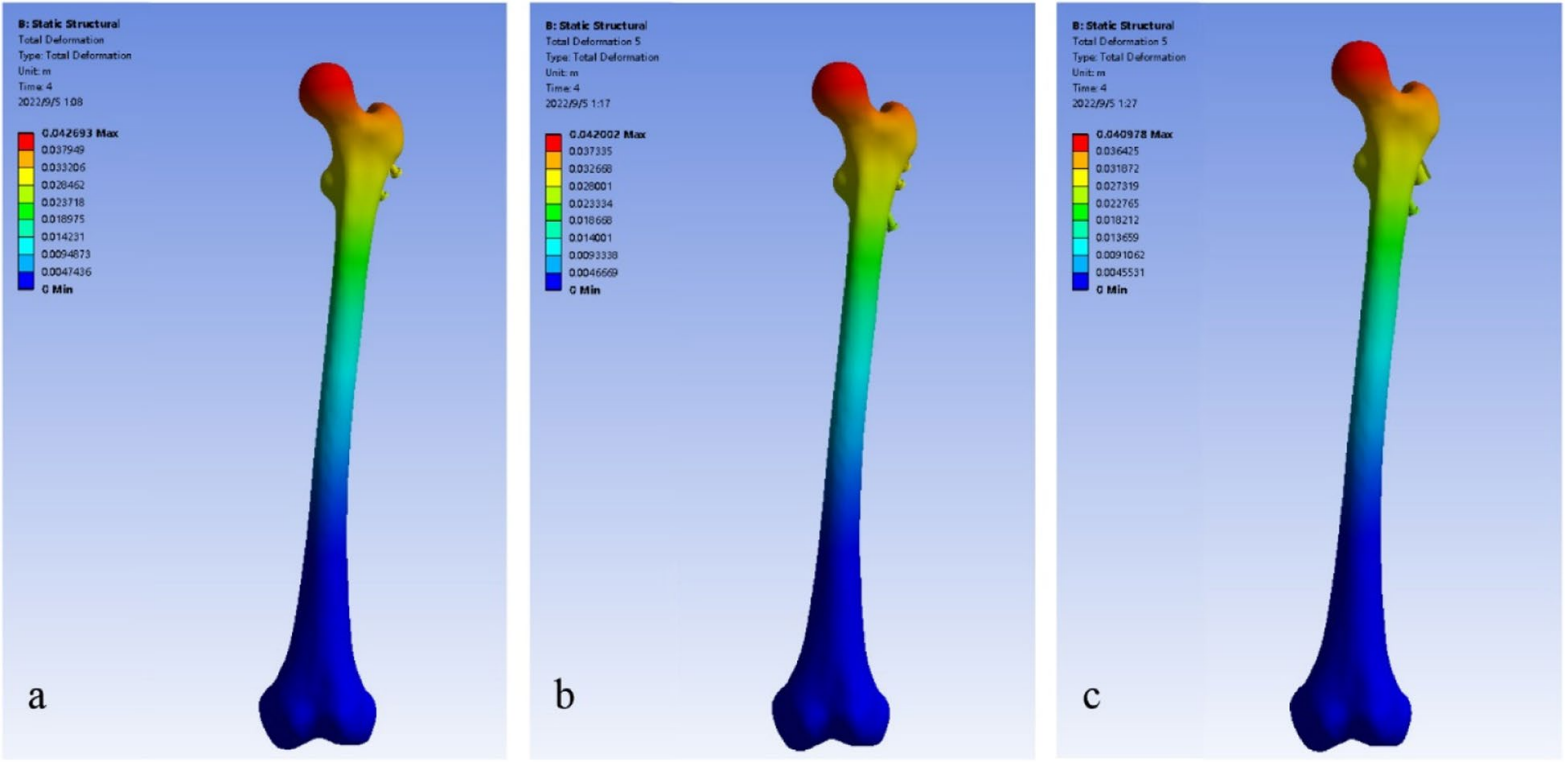
Displacement distributions of femurs at maximum load (**a** 3CS model, **b** BDSF model, **c** SCAB + CS model)

The number of elements and nodes of the models was shown in Table 3. During the loading process, the displacements of three femur models increased with loads (Fig. 3), with the peak displacements always located at the top of femoral heads. The displacements showed a gradually decreasing trend from proximal to distal femur (Fig. 4). Under the maximum loading, the peak displacements of femur were 42.69 mm in model A, 42.00 mm in model B, and 40.98 mm in model C, respectively, seen in Table 4.

**Table 3:** Statistical details of the three finite element models

	Elements	Nodes	Mesh size
Model A	296,340	524,564	1 mm
Model B	310,108	548,542	1 mm
Model C	305,853	539,366	1 mm

**Table 4:** Parameters results measured at maximum load

	Model A	Model B	Model C
Peak displacement of femurs (mm)	42.69	42.00	40.98
Peak Von Mises stress of internal fixations (MPa)	337.75	216.27	205.03
Average Von Mises stress on fracture surfaces (MPa)	12.74	8.73	8.48
Displacement on X-axis of fracture fragments (mm)	21.70	21.39	21.35
Displacement on Z-axis of fracture fragments (mm)	2.81	2.69	2.56

### Von Mises stress distributions of internal fixations

**Fig. 5:**
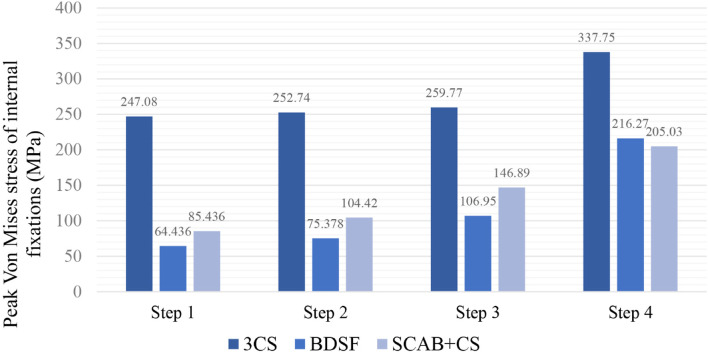
Von Mises stress peaks of internal fixations under increasing loads

**Fig. 6:**
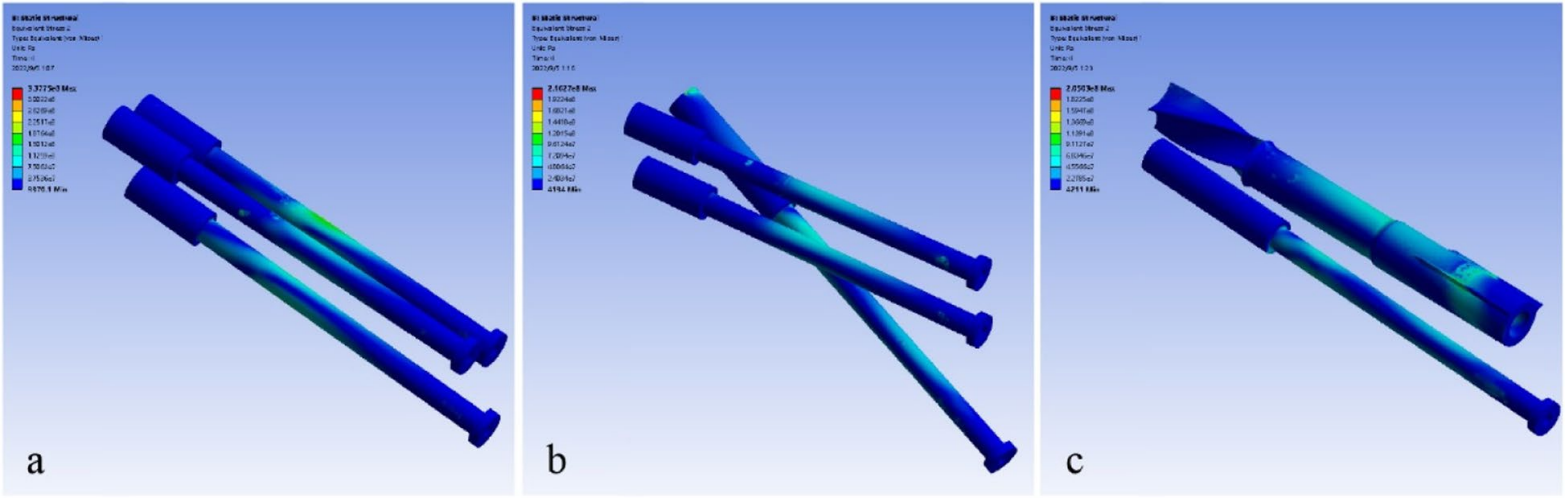
Von Mises stress distributions of internal fixations at maximum load (**a** 3CS model, **b** BDSF model, **c** SCAB + CS model)

The Von Mises stress in three internal fixation models increased with loads. During the growth of loads, the peak Von Mises stress of model A was always much higher than that of the other two models; the peak Von Mises stress of model B was slightly lower than that of model C when the loads were low, while the comparison relationship was reversed at maximum load (Fig. 5). The area with higher Von Mises stress was mainly distributed on the upper and lower surfaces of implants and was dispersed towards the ends with the fracture line as the midpoint, in line with the direction of shearing force (Fig. 6). Under maximum load, the peak Von-Mises stresses were 337.75 MPa for model A, 216.27 MPa for model B and 205.03 MPa for model C, seen in Table 4.

### Von Mises distributions of fracture surfaces at maximum load

**Fig. 7:**
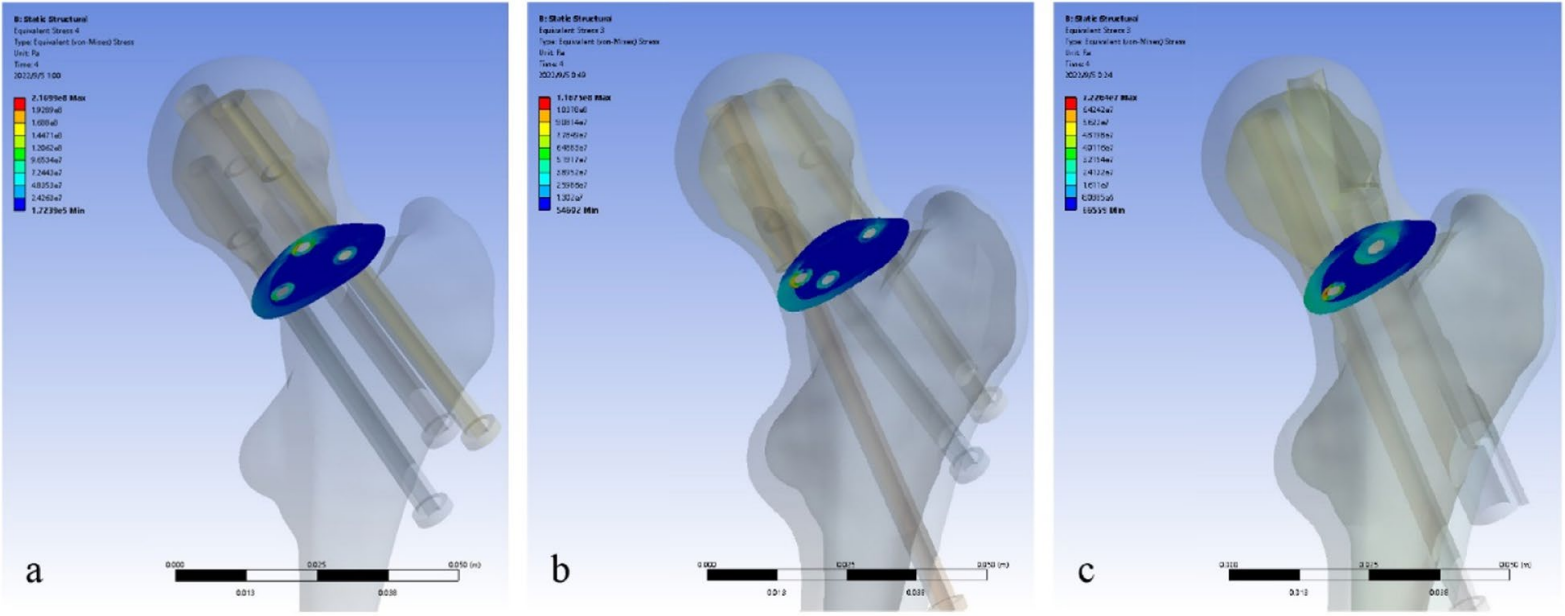
Von Mises stress distribution on fracture surfaces at maximum load (**a** 3CS model, **b** BDSF model, **c** SCAB + CS model)

At maximum load, the average Von-Mises stresses on the fracture surfaces were 12.74 MPa in model A, 8.73 MPa in model B, and 8.48 MPa in model C, respectively. The average Von Mises stress on fracture surface of model A was much higher than that of the other two models (Fig. 7 and Table 4).

### Displacement distributions of fracture fragments at maximum load

**Fig. 8:**
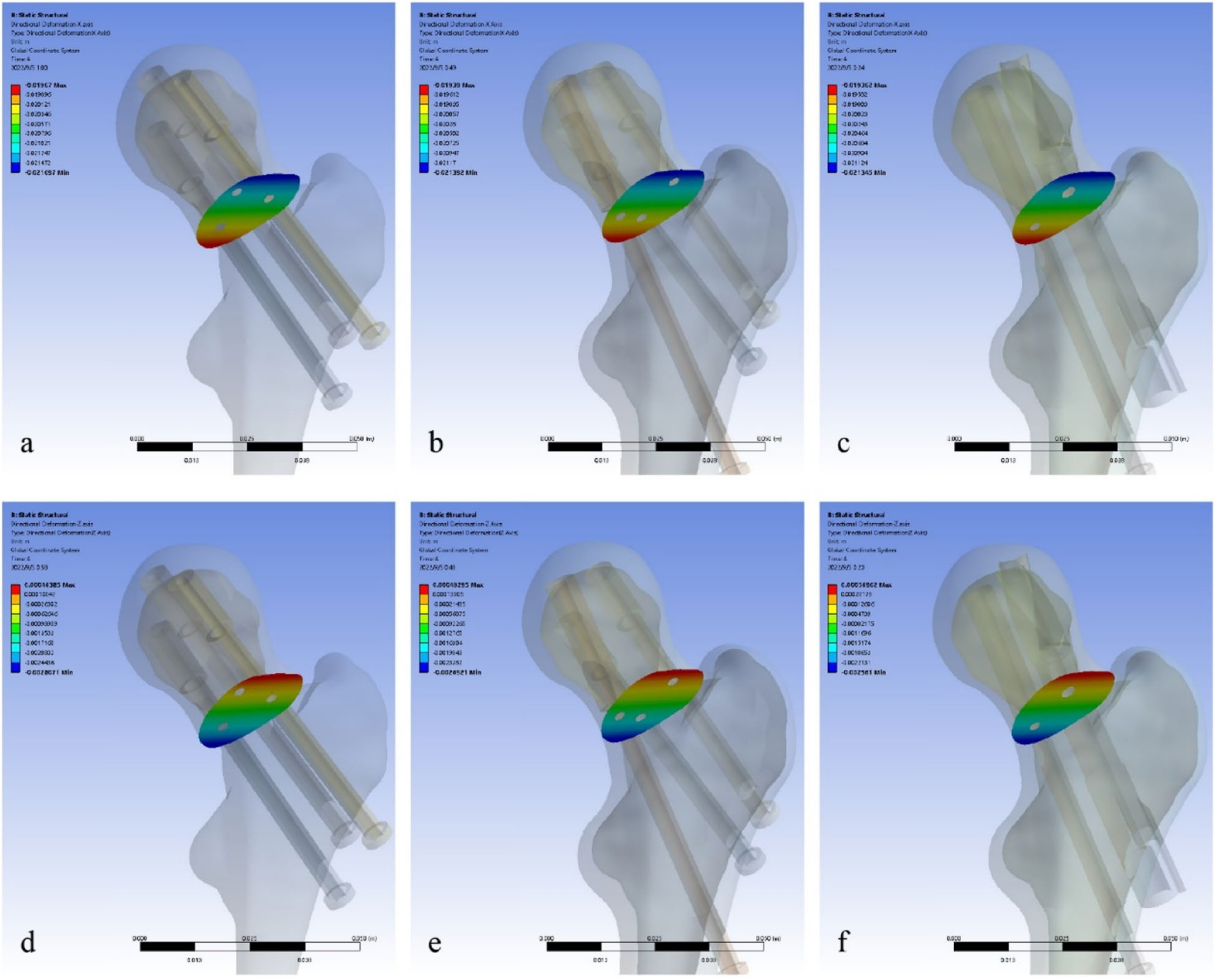
Displacement distributions of proximal fracture fragments on X axis (**a** 3CS model, **b** BDSF model, **c** SCAB + CS model) and on Z axis (**d** 3CS model, **e** BDSF model, **f** SCAB + CS model)

We intercepted the displacement of the proximal fracture fragment on fracture surface and decomposed it into horizontal (X-axis) and vertical (Z-axis) vectors, which can approximately describe the degree of varus displacement and shearing displacement of the femoral heads, respectively. At maximum load, the maximum X-axis displacements of the fracture fragments in models A, B and C were 21.70 mm, 21.39 mm and 21.35 mm, respectively, and the maximum Z-axis displacements were 2.81 mm, 2.69 mm and 2.56 mm, respectively (Fig. 8 and Table 4).

## Discussion

In this study, we compared the biomechanical characteristics of three internal fixation methods, 3CS, BDSF and SCAB + CS, for the treatment of Pauwels type III femoral neck fractures using finite element analyses. The results showed that the SCAB + CS model exhibited lower displacement and Von Mises stress compared to the other two configurations, indicating the stability for internal fixation.

Currently, the optimal internal fixation option for Pauwels type III FNFs is inconclusive and is a hot research issue. In a survey of 272 OTA members on treatment strategies for Pauwels type III FNF in young adults, 47% of specialists opted for fixation with dynamic hip screw and 43% for cannulated screw fixation [30], indicating that there is still a wide divergence in the academic community. Biomechanical studies have shown better mechanical stability with DHS compared to CS [31, 32], but there is a lack of direct evidence of evidence-based medicine proving DHS is related to superior outcomes in clinical practice. The FAITH study in 2017 showed a higher incidence of avascular necrosis of the femoral head after fixation with DHS than fixation with multiple cannulated screws for treating FNFs [33]. A meta-analysis study also pointed out that DHS and CS showed no difference in the rates of reoperation, nonunion and mortality, and CS was superior to DHS on femoral head necrosis [34]. Moreover, DHS has the shortcomings of longer operation time, larger operative trauma, more bleeding, less bone preservation after implantation, and more serious bone damage [6]. In contrast, CS has the advantages of less invasion of soft tissues, less bone damage, simpler procedure and so on, which can well avoid the deficiencies of DHS.

The traditional fixation of 3CS in an inverted triangle configuration has always been controversial in the treatment of unstable FNFs due to poor mechanical stability [35]. Previous studies have shown a high incidence of hip varus deformity and femoral neck shortening after 3CS fixation [23, 36, 37], increasing the risk of internal fixation failure and revision via arthroplasty [38]. The results of our study support previous findings. The 3CS model showed higher displacements of femur and displacements of proximal fracture fragment in both X and Z axis than the other two models, manifesting poor stability, while the internal fixation had the highest peak Von Mises stress, indicating a higher risk of screw break.

Biplane double-supported screw fixation proposed by Filipov [16] improves mechanical properties of internal fixation with CS. This technique adjusts the configuration of three cannulated screws, with the special feature that the distal screw is at a bigger angle with the femoral diaphysis, up to the posterior cortex and touching the femoral calcar. This screw combined with the other two screws forms a dual-support in two planes, capable of withstanding the axial compressive stress and resisting both torsional and shearing stresses [18]. A biomechanical study has shown that the mechanical properties of BDSF are far superior compared to 3CS [39]. In clinical practice, BDSF demonstrated satisfying outcomes as well [17]. Similar findings were obtained in our study. However, our practice experience has shown that placing the distal screw is difficult and may damage the lateral cortex of femoral diaphysis, especially in young patients.

Given the advantages of CS in the treatment of FNFs, we have proposed a new internal fixation method using one SCAB combined with one cannulated screw. According to our design, the helical blade on the head of SCAB can compress the cancellous bone in the femoral head and increase the pull-out force. The diameter of the body of SCAB is much thicker than that of a common cannulated screw, which can better withstand the stresses. The tail fins are arranged in a "V" shape to provide an anti-rotation effect (Fig. 1) [20]. A parallel cannulated screw at the distal end creates a planar configuration with SCAB that further enhances the overall anti-rotation capacity and mechanical stability of the internal fixation. Moreover, the additional cannulated screw effectively distributes the stress, reducing the risk of SCAB breakage and thus internal fixation failure. According to our design, SCAB + CS is indicated for all non-displaced femoral neck fractures (Garden I—II) and displaced femoral neck fractures (Garden III—IV) in non-elderly patients (age < 65 year).

In this finite element analysis study, we adequately simulated the entire postoperative process of a young patient from the initial period to recovery by increasing applied loads [40]. Through observing the displacements of femur, we found that it increased gradually with loads in each model, with the 3CS model consistently showing the highest value, followed by the BDSF model, and the SCAB + CS model being the lowest. At lower loads simulating partial weight bearing, the biomechanical performances of various internal fixation models were similar. However, with increased weight bearing and activity, the SCAB + CS model produced less displacement, therefore we suggest that patients treated with SCAB + CS may be able to undergo functional rehabilitation exercises earlier compared to those treated with 3CS.

Comparing the Von Mises stress distributions of three models, it can be found that the stresses in 3CS model are concentrated on upper surface of cannulated screws near the fracture line, in line with the direction of shearing force, so that the screws are susceptible to top-down bending or breaking. The distal screw in BDSF model withstands more stresses and the maximum Von Mises stress is concentrated on the part of the screw body near the femoral calcar, which is a challenge for the strength of the screw. In SCAB + CS configuration, the stresses are distributed evenly over two implants. The peak stress of SCAB mainly lies on the body and tail pins, and the peak stress of CS is dispersed over the screw. This combination avoids a high concentration of stress in one part of the implant and greatly reduces the risk of internal fixation failure. As the loads gradually increase, the peak Von Mises stress in 3CS model is always much higher than in the other two models, while the peak stress in SCAB + CS model increases less, gradually changing from a slightly higher than that of BDSF model to the lowest of the three models, indicating that the SCAB + CS configuration can effectively disperse the stress and the possibility of mechanical failure is much lower.

We also measured the displacement and Von Mises stress distributions of proximal fracture fragment to assess the ability to prevent complications. The displacements of fracture fragment in both the horizontal and vertical directions were greater in 3CS model than in the remaining two models, suggesting a more pronounced tendency for varus and shearing displacement of the fracture fragment, which also implies that patients treated with 3CS have a higher risk of malunion and non-union than those treated with BDSF or SCAB + CS. As for the stress distribution at the fracture fragment, the SCAB + CS fixation had the lowest result of the three models, the BDSF model was slightly higher, and the 3CS model was much greater than the former two. The lower the stress on the fracture surface, the more mechanical conduction is done by the internal fixation, that is, the more axial support the internal fixation provides, and the less likely the femoral neck shortening resulting from bone absorption will occur.

The novelty of this research lies in the introduction of a new internal fixation method based on SCAB for treating FNFs in young patients, although it is too early to conclude that SCAB + CS is a superior method. There are several limitations to this study. Firstly, the finite element analysis assumes that the object of study is a homogeneous material and sets material properties using uniform modulus of elasticity and Poisson's ratio, which can be somewhat different from the actual condition. In addition, the prognosis of femoral neck fractures treated by internal fixation is closely related to the quality of reduction [23], but our study simulated a fully anatomically repositioned Pauwels type III FNF, which is not always the case in clinical practice. As mentioned above, there are various configurations of 3CS and only traditional inverted triangle configuration included in this study. More comparison with other configurations is needed. Moreover, no experimental validation was conducted, which clearly is a limitation. However, in this study we aimed to observe trends rather than examine the absolute values. In this case, the lack of validation is understandable. In the future, the results of this study are planed to be validated by further in vitro biomechanical tests and clinical trials.

## Conclusion

In this study, a novel internal fixation strategy using SCAB combined with CS for Pauwels type III FNF was introduced. Finite element analyses found that the SCAB + CS internal fixation have potential advantages in terms of stability, mechanical strength, resistance to varus deformity and resistance to shearing force. In conclusion, the novel internal fixation of SCAB combined with CS retains the characteristics of CS in terms of less trauma and ease of operation, and shows satisfied stability and mechanical properties, may providing an alternative for the treatment of unstable vertical femoral neck fractures.

## Data Availability

All the data are available in contact with the corresponding author.

## Abbreviations

FNF: Femoral neck fracture
SCAB: Self-lock compression anti-rotation blade
CS: Cannulated screw
BDSF: Biplane double-supported screw fixation
DHS: Dynamic hip screw
FNS: Femoral neck system
CT: Computed Tomography
DICOM: Digital Imaging and Communications in Medicine
